# Migration of Periodontal Ligament Fibroblasts on Nanometric Topographical Patterns: Influence of Filopodia and Focal Adhesions on Contact Guidance

**DOI:** 10.1371/journal.pone.0015129

**Published:** 2010-12-01

**Authors:** Douglas W. Hamilton, Christine J. Oates, Abdollah Hasanzadeh, Silvia Mittler

**Affiliations:** 1 Graduate Program of Biomedical Engineering, Schulich School of Medicine and Dentistry, The University of Western Ontario, London, Canada; 2 Division of Oral Biology, Schulich School of Medicine and Dentistry, The University of Western Ontario, London, Canada; 3 Department of Physics and Astronomy, The University of Western Ontario, London, Canada; Université de Technologie de Compiégne, France

## Abstract

Considered to be the “holy grail” of dentistry, regeneration of the periodontal ligament in humans remains a major clinical problem. Removal of bacterial biofilms is commonly achieved using EDTA gels or lasers. One side effect of these treatment regimens is the etching of nanotopographies on the surface of the tooth. However, the response of periodontal ligament fibroblasts to such features has received very little attention. Using laser interference lithography, we fabricated precisely defined topographies with continuous or discontinuous nanogrooves to assess the adhesion, spreading and migration of PDL fibroblasts. PDL fibroblasts adhered to and spread on all tested surfaces, with initial spreading and focal adhesion formation slower on discontinuous nanogrooves. Cells had a significantly smaller planar area on both continuous and discontinuous nanogrooves in comparison with cells on non-patterned controls. At 24 h post seeding, cells on both types of nanogrooves were highly elongated parallel to the groove long axis. Time-lapse video microscopy revealed that PDL fibroblast movement was guided on both types of grooves, but migration velocity was not significantly different from cells cultured on non-patterned controls. Analysis of filopodia formation using time-lapse video microscopy and labeling of vinculin and F-actin revealed that on nanogrooves, filopodia were highly aligned at both ends of the cell, but with increasing time filopodia and membrane protrusions developed at the side of the cell perpendicular to the cell long axis. We conclude that periodontal ligament fibroblasts are sensitive to nanotopographical depths of 85–100 µm, which could be utilized in regeneration of the periodontal ligament.

## Introduction

An estimated 50% of the world's population suffers from some form of periodontal disease [1].

This condition is characterized by bacterial infiltration and plaque formation beneath the gingival epithelium against the tooth surface, subsequently resulting in chronic inflammation of the periodontal ligament and gingival tissues [2]. If left untreated, periodontal disease can eventually lead to tooth loss as a direct result of the destruction of the tooth supporting structures (periodontal ligament, gingival connective tissue and alveolar bone) [1], [3], [4]. Of particular importance is the periodontal ligament (PDL) that lines the root of the tooth, functionally linking the tooth with the alveolar bone and allowing dispersal of mechanical forces. When the ligament is damaged the synergy between the bone and tooth is lost and tissue architecture and function becomes significantly disrupted [1].

Current periodontal therapies are aimed at the arrest of periodontal disease progression, and secondarily the regeneration of tissues lost to the disease. Conventional surgical approaches such as flap debridement can reduce periodontal pockets, and slightly enhance the lost periodontal architecture [5]. Of great importance is the removal of biofilms or plaque from the surface of the root [6], which can be done using EDTA gels [7] or erbium-doped:yttrium, aluminum, and garnet (Er:YAG) laser [8]. Such treatments can remove plaque, but secondarily significantly change the topography of the root surface [9], [10], [11], [12], [13]. Commonly, these treatments result in root collagen exposure, dentinal tubule exposure creating surface features in the 50–100 nm range [7]. Interestingly, different treatment regimens (e.g., duration of application) have been shown to vary the size of the topographical features etched on the tooth surface [7], [8]. For regeneration of the PDL adequate cell migration on the tooth root is essential. The topographical features on the root surface will clearly play a critical role in the reattachment of PDL fibroblasts during repair.

Cell-substratum interactions determine many cellular processes such as adhesion, spreading, migration and differentiation [14], [15], [16], [17], [18], processes essential for regeneration of the periodontal ligament. However, the influence of topographical cues on the regulation of PDL fibroblasts has been only sparsely studied. Bruckmann and colleagues demonstrated that microfabricated pit structures (mean diameter of 2.43 µm) resembling dentine tubules did not significantly promote PDL fibroblast attachment, but did increase alkaline phosphatase activity [19]. Apart from the study of Bruckmann et al, the influence of precisely fabricated substratum topography on PDL fibroblast physiology has not been investigated, especially at the nanometric level. As such, the potential benefits of using topographical cues to enhance periodontal regeneration have yet to be utilized. Therefore, we investigated the adhesion, spreading and migration of human PDL fibroblasts in response to continuous and discontinuous topographical cues in the nanometric range. The results from our study suggest that nanometric topographies could be employed to guide and position PDL fibroblasts, a process that will be essential for regeneration of the PDL ligament.

## Materials and Methods

### Surface fabrication

Nanostructures were fabricated as follows. Glass slides were cleaned followed by spin coating of positive photoresist S 1805 (Shipley) at 3000 rpm for 45 seconds. The samples were soft-baked for 30 minutes at 90°C. Using Lloyd's mirror configuration with angle Ø = 19.8° between the mirror and laser beam, the soft baked photoresist on the glass substrates was exposed to the laser.

**Figure 1:**
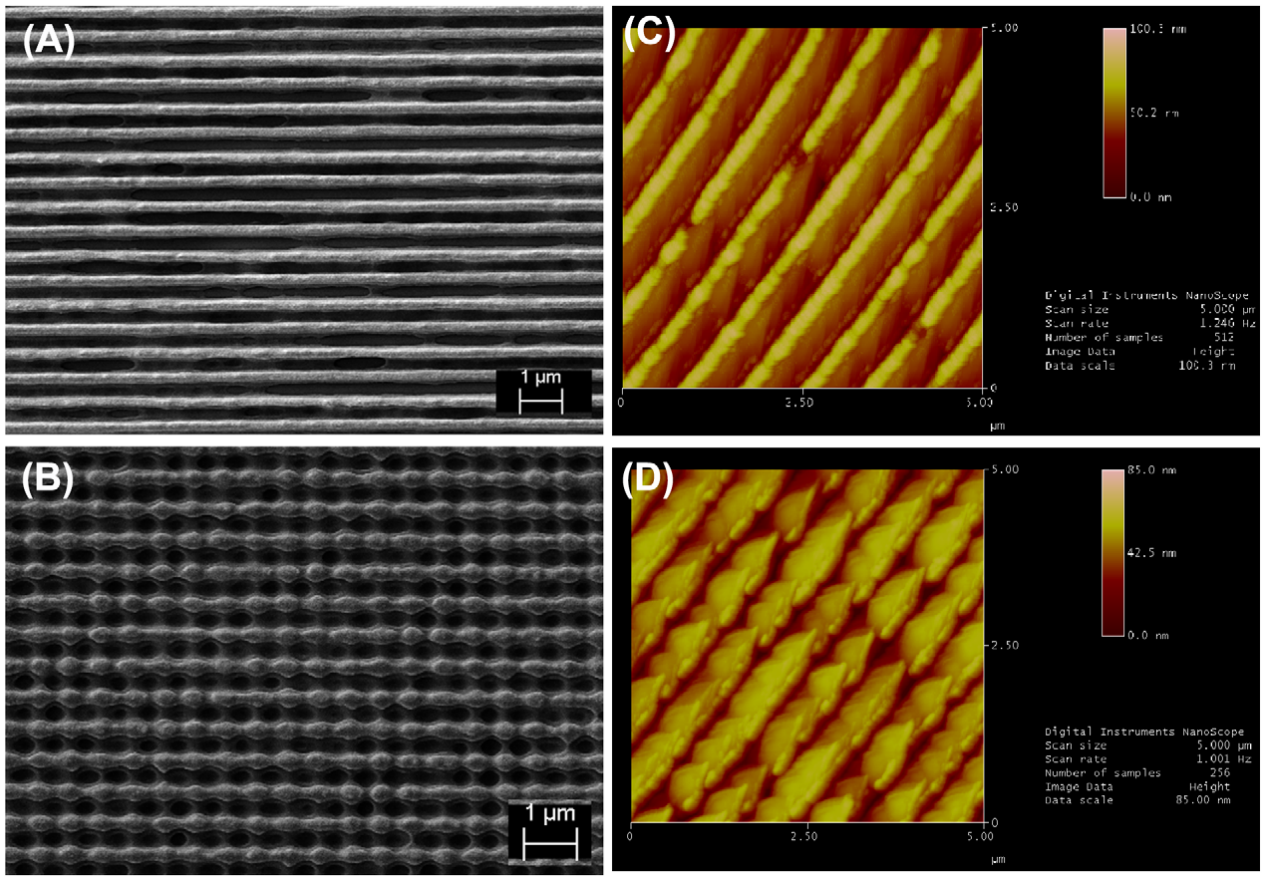
Characterization of the surfaces used in the study. SEM images of the topographies employed in this investigation are shown in (A) nanogrooves, and (B) discontinuous nanogrooves. AFM measurement of (C) nanogrooves – average height 100 nm and (D) discontinuous nanogrooves – average height 85 nm. The pitch of the grooves in both instances was 500 nm. The features were considered to be of suitable dimensions used by previous studies, where 70–100 nm seemed to be a transition point [38].

To make the different patterns, single or double laser exposure of varying time periods were used: single exposure of 30 s (nanogrooves) and for double exposure were 40 s–10 s (discontinuous nanogrooves). Laser intensity in front of the mirror and sample was 80 mW. In the case of the double exposures after the first exposure the sample was rotated by 90 degrees and then the second exposure was done. By developing the exposed parts, the photoresist was removed. In order to stabilize the nanostructures all the samples were hard baked for 30 minutes at 120°C. Argon-gas glow discharge treatment, done prior to seeding with PDL fibroblasts, completed cleaning and sterilization procedures [20]. SEM and AFM images of the surfaces are shown in fig. 1. Single exposures (nanogrooves) resulted in features of 100 nm in depth and double exposures (discontinuous nanogrooves) had features of 85 nm in depth. In both cases, the pitch was 500 nm.

### Atomic Force Microscopy

Atomic force microscopy was performed using a Digital Instruments Multimode AFM with NanoScope IIIa controller (Veeco, Santa Barbara, CA). Measurements were either made in height mode, with a scan direction of 0° (along cantilever axis), while recording the trace and retrace height information (measured in nm), or in lateral-force mode, with a scan direction of 90°, while recording the trace and retrace lateral force data (the A–C signal, measured in V). Scan sizes ranged from 1 to 20 µm, with rates of 0.5 to 4 Hz used to test the dependence on scan speed.

### Isolation and culture of human periodontal ligament fibroblasts

Healthy periodontal ligament was obtained after routine extraction of third molars and premolars in the Oral Surgery Clinic at The University of Western Ontario. The use of this material was in accord with the guidelines of the University's Research Ethics Board for Health Sciences Research involving Human Subjects (HSREB) with informed patient consent. Tissue was harvested from 3 independent patients exhibiting no signs of inflammation in the periodontal tissues (No bleeding, probing depth <3 mm, no visible plaque). Explant cultures were initiated to derive the fibroblastic cells as previously described [21]. PDL fibroblasts were maintained in Dulbecco's modified Eagle's medium (DMEM) supplemented with 10% fetal bovine serum, 100 U/ml penicillin and 100 µg/ml streptomycin at 37°C in a humidified atmosphere of 95% air 5% CO_2_. Cells were removed from the growth surface using a trypsin solution [0.25% trypsin (Gibco), 0.1% glucose, citrate-saline buffer (pH 7.8)] and subsequently used for experiments.

### Adhesion assays

To assess cell number on all surfaces, PDL fibroblasts were seeded at 1,000 cells/cm^2^ and fixed in 10% buffered formalin at 30 min, 2 and 6 h. To calculate cell number, cells were stained with 4′, 6–diamidino-2-phenylindole (DAPI). The samples were then rinsed in 0.1% phosphate buffered saline (PBS) and viewed under ultra-violet optics on a Zeiss fluorescence Axioscope microscope at x 20 magnification using an Axiocam digital camera and AxioImager software (Zeiss). The number of cells per 10 fields of view was calculated from each structure, and data are presented as the mean ± 1 standard error of the mean.

### Cell Spreading Assays

To assess cell spreading on each surface, PDL fibroblasts were seeded at 1,000 cells/cm2 and fixed in 10% buffered formalin at 30 min, 2, 6 and 24 h. Samples were then incubated at room temperature for 90 min with rhodamine-conjugated phalloidin (Invitrogen), and DAPI (Vector Laboratories), respectively. Images from each surface were captured on an AxioScope microscope (Zeiss) using an Axiocam digital camera, and AxioImager software. Cells were thresholded using AxioImager software and the average planar area measured. Data was expressed as average planar area ± standard error of the mean.

### Time-lapse video microscopy

For cell migration assays PDL fibroblasts were seeded on all surfaces at 1,000 cells/cm^2^. Samples were placed in the stage incubator on an Axioscope microscope with phase-contrast optics at a magnification of x10 or x20 at 37°C and 5% CO_2_. Images were captured every 5 minutes for periods of up to 24 h using an Axiocam camera controlled with Axiovision software. Movies were assembled in Axiovison software and were imported into Quicktime (version X, Apple Computers) for analysis and cropping. Montages from each movie were created using Adobe Photoshop 7.0 (Adobe Corp). To calculate cell migration velocity, cells were tracked using the timelapse macro in Axiovision software. Data was imported into Microsoft excel and average velocity and directionality calculated.

### Scanning electron microscopy

Samples were prepared for electron microscopy in triplicate at 24 h post seeding, and were fixed in 2.5% gluteraldehyde in 0.1 M Phosphate buffer (pH 7.2) for 1 hour. Preparation for SEM analysis was performed as previously described [22]. The samples were viewed using a Hitachi 3400-N scanning electron microscope at 4 kV accelerating voltage.

### Immunocytochemistry

For immunofluourescence, PDL fibroblasts were plated at a density of 25,000 cells per structure and cultured for 30 min, 2 h, 6 h, and 24 h, followed by fixation in 4% FA for 10 min, and by three, 5 min washes in PBS. Samples were then stained with a 1∶200 dilution of rhodamine conjugated phalloidin (Sigma-Aldrich), and 1∶100 vinculin (Sigma-Aldrich), followed by FITC linked secondary IgG antibody at a dilution of 1∶200 in 3.0% PBS/Bovine Serum Albumin (BSA) (Molecular Probes Inc./Invitrogen). Samples were counterstained with DAPI to visualize cell nuclei. Images were captured from each surface on an AxioScope microscope using an Axiocam digital camera and AxioImager software. For resizing, images were imported as tiff files into Adobe Photoshop 7.0. Alignment of focal adhesions was calculated using imageJ software. In brief, images of cells labeled for vinculin were thresholded and converted to binary images. Angle of alignment was then calculated relative to the vertical axis.

### Statistical Analysis

For adhesion and experiments, three independent experiments were performed each with 3 replicates for each condition per experiment. Data were analyzed for statistical significance using 2-way analysis of variance (ANOVA) with Bonferroni test (significance assessed at p<0.05) in GraphPad Software v.4 (Graphpad Software, La Jolla, CA) using. In spreading experiments, a minimum of fifteen cells from each treatment group was counted, with 3 replicates per experiment, and three independent experiments performed. Data were analyzed for statistical using 2-way analysis of variance (ANOVA) with Bonferroni test (significance assessed at p<0.05). For migration experiments, 3 independent movies were used for each condition, with statistical significance performed using one-way ANOVA with Tukey's multiple comparison test (significance assessed at p<0.05).

## Results

### Initial Attachment and adhesion formation

**Figure 2:**
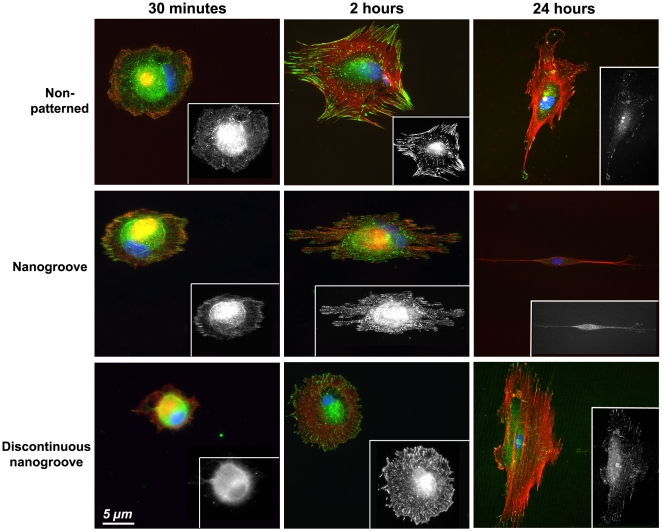
Influence of surface topography on PDL fibroblast attachment, spreading and FA formation 30 min and 2 h after seeding on non-patterned, nanogrooves and discontinuous nanogrooves. Cells were labelled for vinculin (green), F-actin (red) and nucleus (blue). Inset is vinculin staining from the same image to show FA formation alone.

Human PDL fibroblasts attached to all surfaces within 30 min and formed significant focal adhesions by 2 h (fig. 2). On non-patterned surfaces at 30 min, fibroblasts formed numerous punctate adhesions, with F-actin arranged mainly around the periphery of the cells. At 2 h, cells spread extensively characterized by large, mature FAs, with overall cell morphology showing no preferred direction. On nanogrooves at 30 min, highly aligned FAs were evident at the periphery of the cells, parallel to the groove long axis, but no obvious F-actin stressfibres were observed to have formed. At 30 min on discontinuous nanogrooves, no FAs were evident with vinculin immunoreactivity distributed throughout the cytoplasm. However, at 2 h on discontinuous nanogrooves, cells formed FAs, but overall morphology showed no preferred orientation in relation to the grooves.

### Quantification of Attachment Spreading and Focal Adhesion Alignment

**Figure 3:**
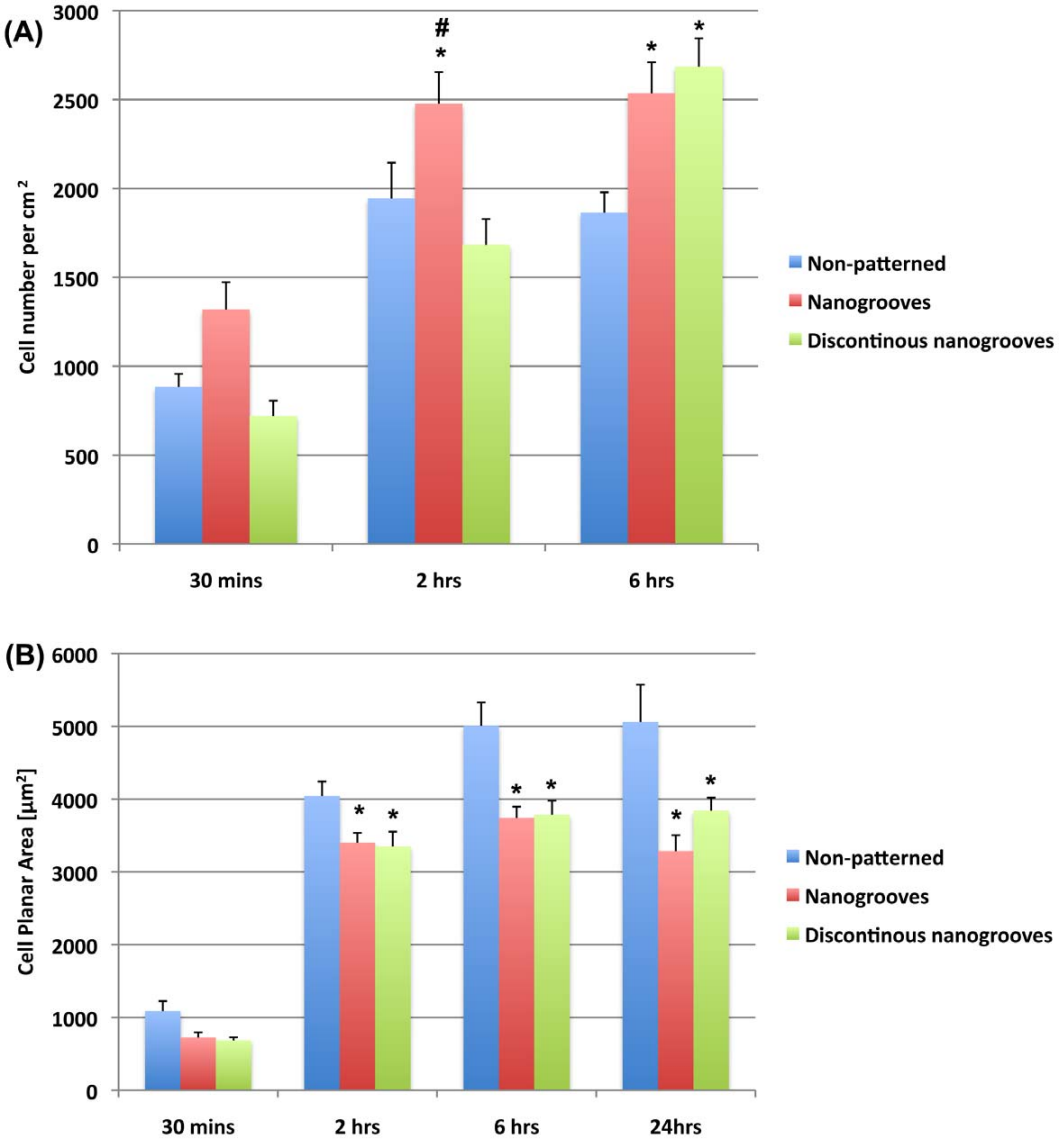
Quantification of PDL fibroblast (A) adhesion, and (B) spreading, on non-patterned surfaces, nanogrooves, and discontinuous nanogrooves. In (A). *  =  significantly different from non-patterned (p<0.05) and #  =  statistically significant from discontinuous nanogrooves (p<0.05).

**Figure 4:**
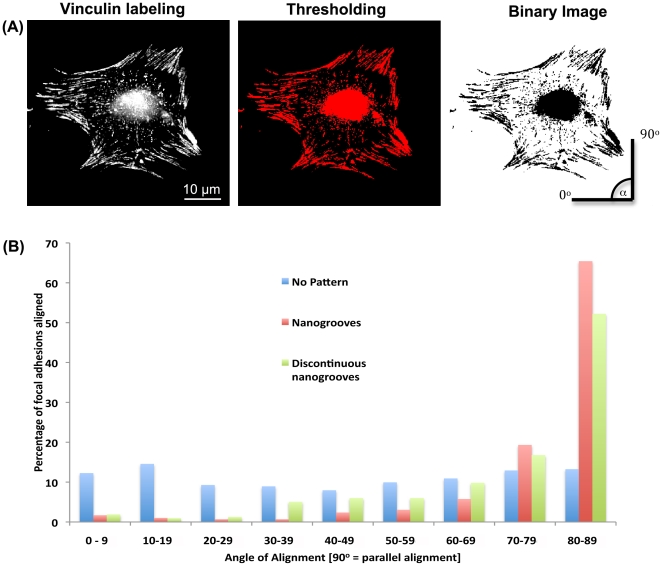
Influence of continuous and discontinuous nanogrooves on alignment of focal adhesions in PDL fibroblasts. (A) images of cells labeled for vinculin were thresholded and converted to binary images. Angle of alignment was then calculated relative to the vertical axis. (B) distribution of focal adhesion alignment.

**Figure 5:**
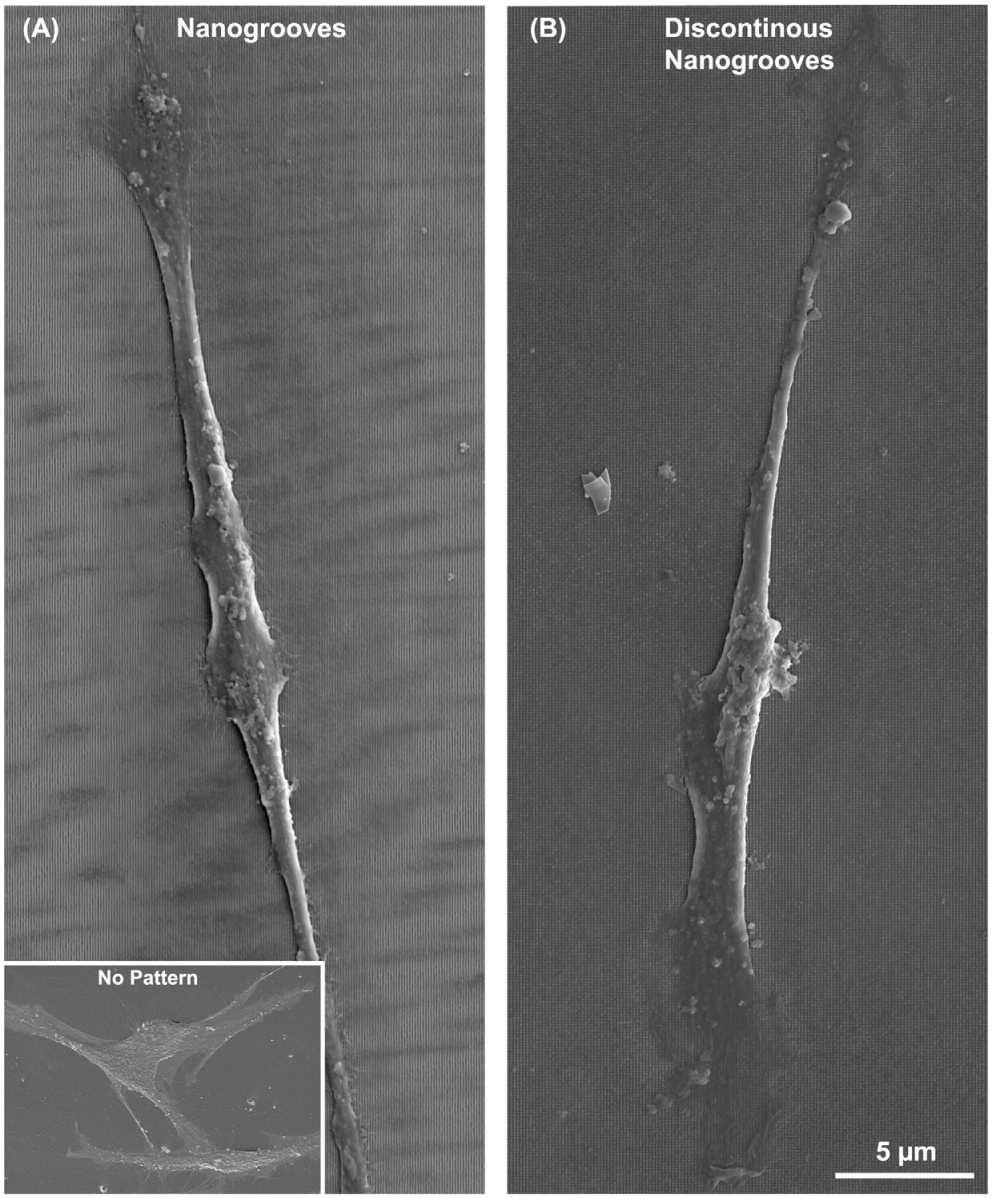
Scanning electron micrographs of PDL fibroblasts cultured on (A) nanogrooves, and (B) discontinuous nanogrooves 24 h post seeding. Inset shows PDL fibroblasts grown for 24 h on non-patterned surfaces.

Quantification of cell attachment demonstrated that significantly more cells were attached to

nanogrooves at 30 min and 2 h post seeding than any other surface (fig. 3A). At 6 h post seeding, significantly more cells were attached to nanogrooves and discontinuous nanogrooves in comparison with non-patterned control (p<0.05). Analysis of spreading demonstrated that cell planar area was significantly higher on smooth surfaces at 2, 6 and 24 h post seeding in comparison with nanogrooves and discontinuous nanogrooves (p<0.05) (fig. 3B). Cells cultured on nanogrooves and discontinuous nanogrooves were significantly smaller at 24 h post seeding compared to cells cultured non-patterned surfaces (p<0.05). Analysis of FA alignment at 24 h post seeding demonstrated that cells on continuous and discontinuous nanogrooves exhibited a high level of alignment in comparison with controls (fig. 4). Similarly, SEM analysis of cells at 24 h demonstrated that on smooth surfaces, cells showed no preferred direction, but on nanogrooves and discontinuous nanogrooves, cells were highly elongated parallel with the groove long axis (fig. 5).

### Migration

**Figure 6:**
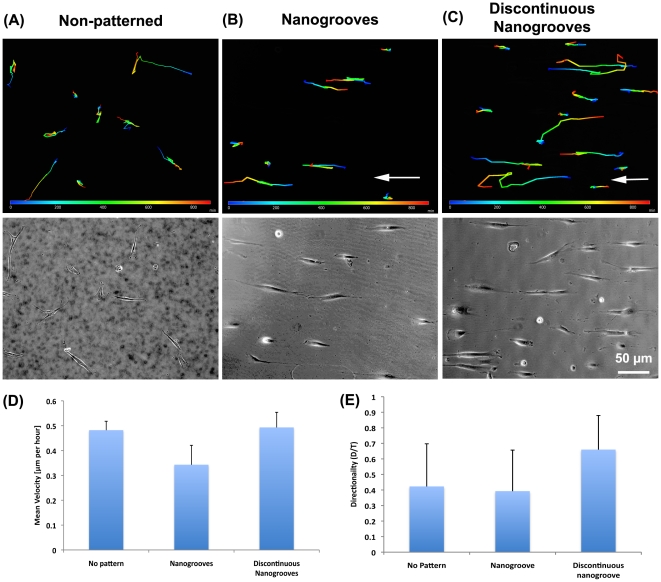
Analysis of PDL fibroblast migration on (A) smooth, (B) nanogrooves, and (C) discontinuous nanogrooves (see also supplementary movies S1, S2, and S3). The top panel in all images shows the cell tracking patterns for each movie. Arrow indicates orientation of the nanotopographies long axis. In (D) quantification of PDL fibroblast migration velocity on each tested topography, and in (E) influence of topography on directionality of the PDL fibroblast migration patterns. Data was analyzed using Axioimager software and statistical analysis was performed using one-way ANOVA with Bonferroni post-hoc test.

To assess the ability of fibroblasts to migrate on all types of topographies, time-lapse video microscopy was performed. On non-patterned surfaces, cells were observed to show no preferred direction of movement (fig. 6A and movie S1). On nanogrooves, cells had an elongated morphology and migrated parallel to the groove long axis (fig. 6B and movie S2). Similarly, cells cultured on discontinuous nanogrooves were highly oriented and migrated in the direction of the groove axis (fig. 6C and movie S3). Quantification of cell migration velocity demonstrated that cells did not move significantly faster on any of the nanotopographies in comparison with smooth controls (fig. 6D), and no significant differences in directionality were observed (fig. 6E).

### Morphological changes during cell migration

**Figure 7:**
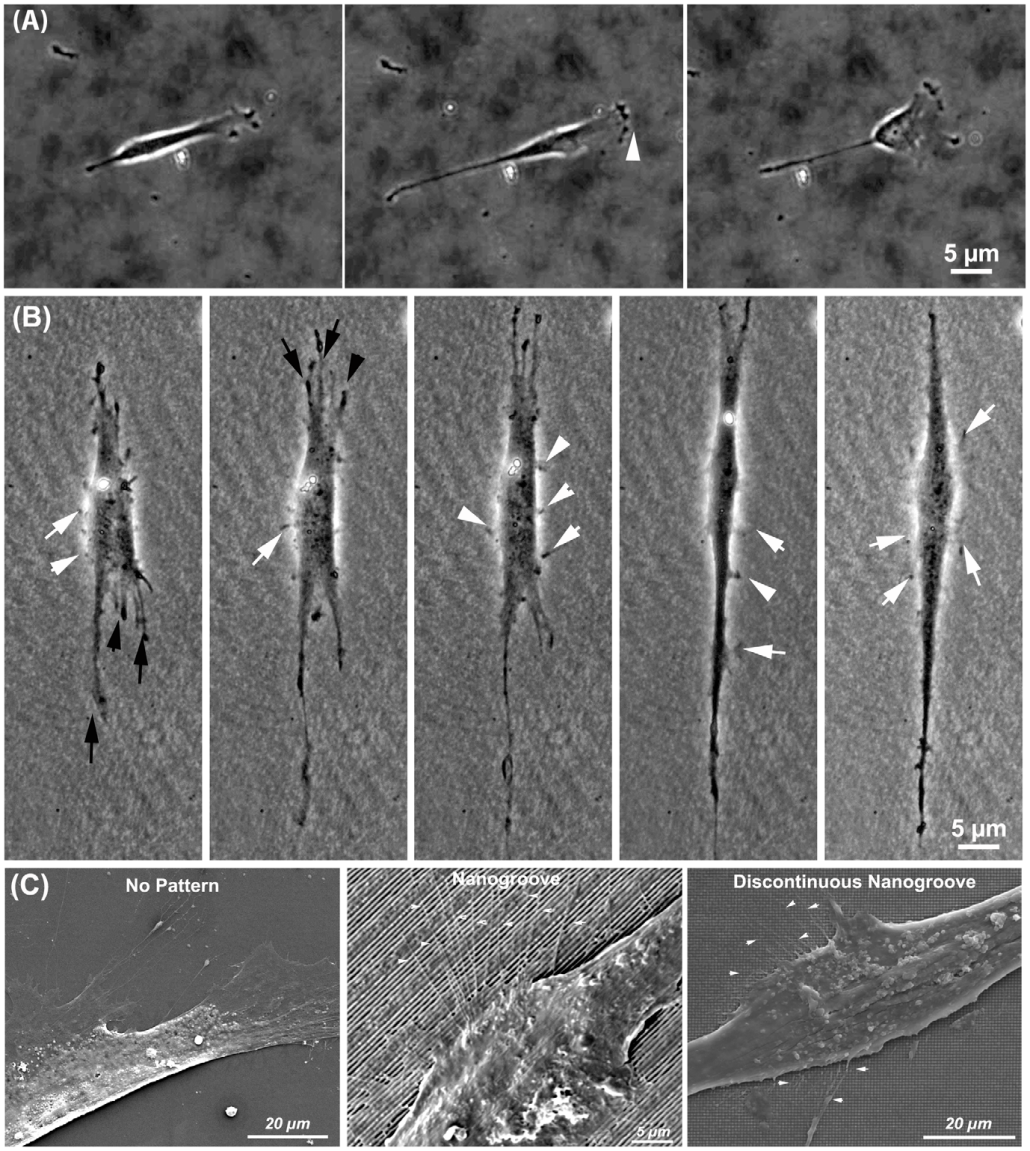
Sequential timelapse images of PDL fibroblast spreading on (A) non-patterned surfaces and (B) nanogrooves. Images in (B) can be seen also in supplementary movie S4. In (C), scanning electron micrographs of filopodia formation in PDL fibroblasts cultured on nonpatterned, nanogrooves and discontinuous nanogrooves. In (A) arrowhead shows the direction of lamelipodia extension. In (B), arrows show filopodia and membrane protrusion extending along the length of the fibroblast during spreading.

To compare how PDL fibroblasts migrated on nanotopographies, cells were analyzed at high magnification (fig. 7 and movie S4). On non-patterned surfaces, cells formed a prominent leading lamelipodia, the cell elongated, then the trailing end of the cell retracted and the cell moved forward (fig. 7A). On nanogrooves, as cells began to spread, numerous cell processes were evident at both ends of the cell, with filopodium extended at the cell periphery (fig. 7B). Certain extensions at the end of the cells persisted, with the direction of movement parallel to the groove long axis. As the cells extended, numerous extensions appeared attaching to the topography along the length of the cell body, almost in a wave-like pattern. Such protrusions were short in duration and did not influence the overall direction of cell spreading. At the ends of the cells, eventually one filopodium would dominate as the cell continued to elongate along the grooves. Protrusion of cell extensions continued to persist at the edge of the cell body (fig. 7C).

### Adhesion formation and cytoskeletal changes during migration

**Figure 8:**
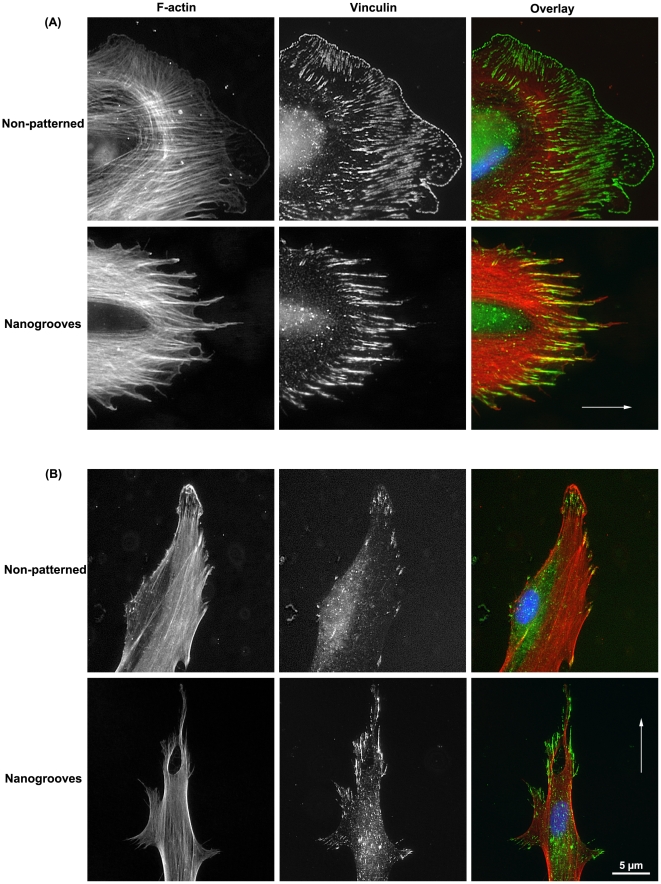
Analysis of vinculin (green) and F-actin (red) localization in filopodia extended by PDL fibroblasts cultured on smooth surfaces and nanogrooves at (A) 2 h and (B) 24 h. Arrows indicate direction of groove long axis.

As cell spread on non-patterned surfaces up to 2 h, a well-developed leading lamelipodium was observed at the front of the cell, with a hierarchical arrangement of FA present (immature at the cell edge, mature towards the centre of the cell) (fig. 8A). F-actin stressfibres predominantly oriented to the direction of lamelipodia extension, although other bundles were arranged circumferentially (fig 8A). On nanogrooves, both FA and F-actin were well aligned with the groove long axis and numerous well-developed filopodia were observed (fig. 8A). 6 h post seeding, on non-patterned surfaces, cells were observed to have both leading lamelipodia and filopodia, the latter aligned in the same direction as the lamelipodia (fig. 8B). These were also the prominent sites of vinculin containing FAs, with F-actin stressfibers extending the length of the cells. On nanogrooves at 6 h, cells were well oriented to the groove long axis, with larger FAs prominent at the ends of the cells. Assessment of cell extensions at the side of the cell revealed numerous fine filopodia along with larger protrusions of the membrane (fig. 8B). Dual labeling of protrusions for F-actin and vinculin revealed significant FAs and well-aligned F-actin stressfibres, perpendicular to the long axis of the cell (fig. 8B).

## Discussion

The adhesion and migration of periodontal ligament fibroblasts on the root surface is essential for regeneration of the periodontal ligament [23]. Nanometric topographies are commonly etched on teeth incidentally during the removal of dental plaque [7]. The use of topographical cues to guide cells into specified positions (contact guidance) has been used with great effect around dental implants [24], [25], [26], [27], but the role of contact guidance in PDL regeneration around natural teeth is largely unknown. Moreover, little research on PDL fibroblast response to topographical cues has been performed. Therefore, we investigated how precisely defined nanotopographical patterns influence the migration of human PDL fibroblasts *in vitro*.

The first step in contact guidance of cells on nanogrooves is adhesion and guided spreading, typically with the cell assuming an elongated morphology parallel to the groove long axis [28], [29]. The exact mechanisms that result in cell alignment on nanotopographies such as grooves remain controversial, but likely involve the extension of filopodia [15], [30]. Dermal fibroblasts align to grooves as shallow as 35 nm if cultured for sufficient time, below which alignment of cells no longer appears to exist [31]. Dalby et al 2006, demonstrated that osteoprogenitor cells aligned focal adhesions as well as overall cell morphology on grooves of 5 µm pitch and 510 nm depth [32]. In contrast, Teixiera et al 2003, stated that alignment of focal adhesions is not a pre-requisite for epithelial cell contact guidance on nanogrooves with 70 nm wide ridges [15], Moreover, they demonstrated that on nanogrooves below 800 nm depth, epithelial cells did not contain aligned FAs [33]. We noted differences in initial adhesion and spreading on each tested surface (fig. 2, 3). On 100 nm deep, 500 nm pitch nanogrooves, FAs were well aligned as early as 30 mins post seeding, and by 2 h overall cell morphology was aligning in the direction of the groove long axis. In contrast, we observed that the presence of discontinuities on 85 nm depth (500 nm pitch) nanogrooves inhibited alignment of both FAs and cells at 2 h. Therefore, it appears that alignment of FAs is a pre-requisite for PDL fibroblast alignment on nanotopographies. Possible explanations for the differences in requirement of FA alignment could be related to the cell type (epithelial vs fibroblast). We have previously shown that osteoblasts and epithelial cells adhere, spread and migrate in a very different manner on the same truncated pit micro-topographies [34]. Moreover, it is important to take into account that contact guidance on fine nanofeatures is likely influenced by other factors such as compression, mechanical stress [31] or potentially even the culture conditions [33].

Although alignment of FAs and cell morphology is part of contact guidance, typically the overall process is considered the directed migration of a cell in a specified direction by a physical cue [29], [35], [36]. Of the cellular processes influenced by nanotopographies, analysis of cell migration has probably received the least attention and many studies are based on observation of fixed cells [30], [31], [37], [38], [39]. In our study, using timelapse video microscopy, we show that PDL fibroblasts do not move significantly faster on either nanogrooves or discontinuous nanogrooves in comparison with cells cultured on non-patterned surfaces, nor do they exhibit significant changes in directionality. This is in direct contrast with micron-scale grooves, where the migration rates and directionality of many different cell types increases significantly in comparison with cells cultured on non-patterned surfaces [39], [40], [41]. In normal cell migration models on 2 dimensional surfaces, cells form a leading lamellipodia from which filopodia extend, guiding the direction of movement. Within the lamelipodia, the cell forms adhesions to the substratum, which allow the cell to gain traction. However, it was noted in our study that as PDL fibroblasts spread and aligned on nanotopographies, they were observed to form numerous vinculin-rich filopodia at both ends of the cell (evident in figs. 2 and 7). We propose that the lack of a dominant leading lamelipodium reduces the migration velocity as the ends of the cells become involved in a “tug-of-war” over dominance. No true lamelipodium typically persists, with ends of the cells elongating until similar in appearance to filopodium (fig. 7). As the topography at both ends of the cell is identical, it appears that both ends of the cell attempt to be dominant in migration (S4). When one end of the cells forms enough FAs to generate traction, it likely results in guided migration in that direction through retraction of the other end of the cell.

When cells attach to a surface, filopodia are the initial mechanisms through which a cell “senses” its surroundings and it has been well established that cells extend filopodia at or near nanotopographical cues such as grooves and nanocolumns [15], [30], [42]. Dalby et al, 2005, demonstrated that cells would extend filopodia to structures as small as 35 nm in depth, although nanocolumns of 75 nm or higher elicited a more obvious response from cells [30]. During our analysis of cell migration using timelapse video microscopy, we observed that cells extend numerous filopodia along the length of the cell body on both nanogrooves and discontinuous nanogrooves (fig. 6 and 7). As no previous studies have shown timelapse video microscopy to analyze the filopodia extensions, understanding how persistent these structures are on nanotopographies has never been assessed. We show here for the first time that these filopodia are continually forming and often develop into small lamelipodia, which persist over short periods of time. Interestingly, these extensions do not appear to strongly influence the overall alignment of the cell with the topographical pattern. We conclude that even although cells align with nanogrooves, they continue to explore the surrounding topography without changing alignment. Future studies will focus on whether inhibition of filopodia formation prevents PDL fibroblast alignment to nanotopographies.

While PDL fibroblasts are clearly responsive to nanofeatures, not all nanotopographies promote adhesion and certain geometries inhibit cell attachment and growth [43], [44]. In our study, we employed both continuous and discontinuous nanogrooves, and although similar cell response were evident on both topographical types, distinct differences were evident in spreading and FA formation at early timepoints. We have previously shown that discontinuous topographies are a powerful modulator of FA formation, cytoskeletal organization and fibroblast migration [45], [46]. Re-establishing the PDL architecture after degeneration would require active migration of the cells on the tooth surface and the results of our study suggest that both continuous and discontinuous nanometric topographical cues could be employed to facilitate this process. Therefore, more emphasis should be placed on the root surface and its topography in attempts to regenerate the periodontal ligament structure after treatment of gingivitis or periodontitis. Moreover, such features could also be added to guided tissue regeneration membranes used in regeneration of the periodontal structures after surgical intervention.

## Supporting Information

Movie S1 **Timelapse video of PDL fibroblast migration on non-patterned surfaces.** 1 second  = 1 hour of actual time.
(MOV)

Movie S2 **Timelapse video of PDL fibroblast migration on nanogrooves of 100 nm depth and 500 nm pitch.** 1 second  = 1 hour of actual time.
(MOV)

Movie S3 **Timelapse video of PDL fibroblast migration on discontinuous nanogrooves of 85 nm depth, 500 nm pitch.** 1 second  = 1 hour of actual time.
(MOV)

Movie S4 **Timelapse video of PDL fibroblast migration on nanogrooves of 100 nm depth and 500 nm pitch.** 1 second  = 1 hour of actual time.
(MOV)
